# Morocco's First Biobank: Establishment, Ethical Issues, Biomedical Research Opportunities, and Challenges

**DOI:** 10.1155/2020/8812609

**Published:** 2020-12-08

**Authors:** Saida Lhousni, Karam Yahya Belmokhtar, Ihab Belmokhtar, Mounia Elidrissi Errahhali, Manal Elidrissi Errahhali, Redouane Boulouiz, Mariam Tajir, Majida Charif, Khawla Zerrouki, Noufissa Benajiba, Maria Rkain, Abdeladim Babakhouya, Hatim Kouismi, Afaf Thouil, Hanane Latrach, Rim Amrani, Sahar Messaoudi, Anass Ayyad, Zaina Sidqi, Khalid Andaloussi Serraj, Siham Hamaz, Habiba Alaoui, Houda Bachir, Yassamine Bentata, Intissar Haddiya, Mohammed Choukri, Rachid Seddik, Amal Bennani, Siham Dikhaye, Bouchra Oneib, Fatima Elghazouani, Omar El Mahi, Adnane Benzirar, Ayat Allah Oufkir, Brahim Housni, Ahmed Mimouni, Hanane Saadi, Mohammed Belahcen, Tijani El Harroudi, Meryem Ouarzane, Mohammed Bellaoui

**Affiliations:** ^1^Genetics Unit, Medical Sciences Research Laboratory, Faculty of Medicine and Pharmacy, University Mohammed Premier, Oujda, Morocco; ^2^Higher Institute of Nursing Professions and Health Technologies, Oujda, Morocco; ^3^Medical Genetics Unit, Central Laboratory, Mohammed VI University Hospital, Faculty of Medicine and Pharmacy, University Mohammed Premier, Oujda, Morocco; ^4^Genetics, and Immuno-Cell Therapy Team, Faculty of Sciences, University Mohammed Premier, Oujda, Morocco; ^5^Department of Pediatrics, Mohammed VI University Hospital, Faculty of Medicine and Pharmacy, University Mohammed Premier, Oujda, Morocco; ^6^Department of Pulmonology, Mohammed VI University Hospital, Faculty of Medicine and Pharmacy, University Mohammed Premier, Oujda, Morocco; ^7^Department of Endocrinology, Mohammed VI University Hospital, Faculty of Medicine and Pharmacy, University Mohammed Premier, Oujda, Morocco; ^8^Department of Neonatology, Mohammed VI University Hospital, Faculty of Medicine and Pharmacy, University Mohammed Premier, Oujda, Morocco; ^9^Transfusion Regional Centre, Oujda, Morocco; ^10^Department of Internal Medicine, Mohammed VI University Hospital, Faculty of Medicine and Pharmacy, University Mohammed Premier, Oujda, Morocco; ^11^Nephrology and Kidney Transplantation Unit, Mohammed VI Faculty of Medicine and Pharmacy, University Hospital, University Mohammed Premier, Oujda, Morocco; ^12^Biochemistry Unit, Central Laboratory, Mohammed VI University Hospital, Faculty of Medicine and Pharmacy, University Mohammed Premier, Oujda, Morocco; ^13^Hematology Unit, Central Laboratory, Mohammed VI University Hospital, Faculty of Medicine and Pharmacy, University Mohammed Premier, Oujda, Morocco; ^14^Pathology Unit, Central Laboratory, Mohammed VI University Hospital, Faculty of Medicine and Pharmacy, University Mohammed Premier, Oujda, Morocco; ^15^Department of Dermatology, Mohammed VI University Hospital, Faculty of Medicine and Pharmacy, University Mohammed Premier, Oujda, Morocco; ^16^Department of Psychiatry, Mohammed VI University Hospital, Faculty of Medicine and Pharmacy, University Mohammed Premier, Oujda, Morocco; ^17^Department of Vascular Surgery, Mohammed VI University Hospital, Faculty of Medicine and Pharmacy, University Mohammed Premier, Oujda, Morocco; ^18^Department of Plastic Surgery, Mohammed VI University Hospital, Faculty of Medicine and Pharmacy, University Mohammed Premier, Oujda, Morocco; ^19^Department of Anesthesiology and Reanimation, Mohammed VI University Hospital, Faculty of Medicine and Pharmacy, University Mohammed Premier, Oujda, Morocco; ^20^Department of Gynecology and Obstetrics, Mohammed VI University Hospital, Faculty of Medicine and Pharmacy, University Mohammed Premier, Oujda, Morocco; ^21^Department of Pediatrics Surgery, Mohammed VI University Hospital, Faculty of Medicine and Pharmacy, University Mohammed Premier, Oujda, Morocco; ^22^Department of Surgical Oncology, Mohammed VI University Hospital, Faculty of Medicine and Pharmacy, University Mohammed Premier, Oujda, Morocco; ^23^Faculty of Sciences, University Mohammed Premier, Oujda, Morocco

## Abstract

**Background:**

Biobanks are highly organized infrastructures that allow the storage of human biological specimens associated with donors' personal and clinical data. These infrastructures play a key role in the development of translational medical research. In this context, we launched, in November 2015, the first biobank in Morocco (BRO Biobank) in order to promote biomedical research and provide opportunities to include Moroccan and North African ethnic groups in international biomedical studies. Here, we present the setup and the sample characteristics of BRO Biobank.

**Methods:**

Patients were recruited at several departments of two major health-care centers in the city of Oujda. Healthy donors were enrolled during blood donation campaigns all over Eastern Morocco. From each participant, personal, clinical, and biomedical data were collected, and several biospecimens were stored. Standard operating procedures have been established in accordance with international guidelines on human biobanks.

**Results:**

Between November 2015 and July 2020, 2446 participants were recruited into the BRO Biobank, of whom 2013 were healthy donors, and 433 were patients. For healthy donors, the median age was 35 years with a range between 18 and 65 years and the consanguinity rate was 28.96%. For patients, the median age was 11 years with a range between 1 day and 83 years. Among these patients, 55% had rare diseases (hemoglobinopathies, intellectual disabilities, disorders of sex differentiation, myopathies, etc.), 13% had lung cancer, 4% suffered from hematological neoplasms, 3% were from the kidney transplantation project, and 25% had unknown diagnoses. The BRO Biobank has collected 5092 biospecimens, including blood, white blood cells, plasma, serum, urine, frozen tissue, FFPE tissue, and nucleic acids. A sample quality control has been implemented and suggested that samples of the BRO Biobank are of high quality and therefore suitable for high-throughput nucleic acid analysis.

**Conclusions:**

The BRO Biobank is the largest sample collection in Morocco, and it is ready to provide samples to national and international research projects. Therefore, the BRO Biobank is a valuable resource for advancing translational medical research.

## 1. Introduction

A biobank is a highly organized structure that stores human biological samples associated with the donors' medical and personal information. The last several decades have seen a dynamic development of biobanks. Indeed, hundreds of biobanks have been created in many countries to provide thousands of human biospecimens from patients and/or healthy individuals to research programs. These resources have become indispensable tools for biomedical research [1, 2]. Indeed, biobanks have been used by researchers to advance treatment and prevention of many diseases and to develop innovative diagnostic tests [3–7].

Biobanking should harmonize a number of steps: collection of biological samples (tissues, blood, urine, etc.), processing, storage, and quality control of these samples; management of medical information; and finally, providing them to research projects. Therefore, biobanking requires coordinated action among many stakeholders such as donors, clinicians, nurses, laboratory technicians, scientists, healthcare providers, and ethicists [8].

A major challenge for biobanking is to ensure that the stored samples are well preserved and of high quality. Another challenge is to make these samples easily accessible to researchers leading to the generation of reliable research data accepted by the scientific community. Therefore, standard operating procedures (SOPs) describing all aspects of biobanking (sample collection, processing, storage, and quality control) have been established by various biobank organizations, such as the International Society for Biological and Environmental Repositories (ISBER), the National Cancer Institute (NCI), OECD Recommendation on Human Biobanks and Genetic Research Databases, and the European, Middle Eastern and African Society for Biobanking and Biopreservation (ESBB) [9–15].

Although biobanks have been in place in high-income countries for many years; it is only recently that they have been implemented in some low- and middle-income countries [16–19]. In this context, we launched in November 2015 a biobank in the eastern region of Morocco (BRO Biobank) in order to promote biomedical research in Morocco where research resources are limited. In this paper, we present all details of the creation of the BRO Biobank (ethical aspect, patient and healthy donor recruitment, processing, storage and quality control of the biospecimens, and management of medical information). We also present the sample collection of the BRO Biobank. We then discuss the challenges of the BRO Biobank.

## 2. Materials and Methods

### 2.1. Ethical Considerations

In Morocco, there is currently no specific law on biobanks or legislation addressing biomedical research. However, there is a law on the protection of individuals with regard to the processing of personal data [20]. Therefore, in accordance with this law, which is relevant for biomedical research and biobanking, we have established an ethical protocol for the BRO Biobank. We have also taken into account the laws and ethical guidelines for biobanks or biomedical research involving human subjects from different high-income countries such as Belgium, Finland, France, Iceland, and Germany [21–25].

The BRO Biobank's ethical protocol was approved by the Ethical Review Committee for Biomedical Research of the Faculty of Medicine and Pharmacy of Casablanca (CERBC). The authorization for personal data processing was obtained from the data protection agency of Morocco (CNDP). The main ethical issues addressed in the BRO Biobank's ethical protocol were confidentiality, informed consent, secondary use of samples and data over time, use of genetic material, children's participation in biobanks, return of results to participants, and sharing of data and samples with other scientists. Written informed consent was obtained from each participant at enrolment.

### 2.2. BRO Biobank's Facility

The BRO Biobank is located at the Genetics Unit of the Faculty of Medicine and Pharmacy of Oujda. The Genetics Unit is organized into a cytogenetic lab, a molecular biology lab, and a room with several freezers for storing samples.

### 2.3. Patients and Healthy Donors

Patients were recruited into the BRO Biobank at several medical and chirurgical departments of two major health care centers in the city of Oujda (Mohammed VI University Hospital Center and El Farabi Regional Hospital). Regarding healthy individuals, recruitment into the BRO Biobank was carried out at the Regional Blood Transfusion Center of Oujda and during the blood donation campaigns in the other cities of Eastern Morocco. Patients or healthy donors were approached and given explanations about the purpose of biobanking. They were then invited to participate in the BRO Biobank. Individuals who agreed to participate in the BRO Biobank were asked to sign an informed consent available in Arabic and French.

### 2.4. Collection, Transport, and Reception of Biospecimens at the BRO Biobank

Blood or tissue samples (surgical specimens or biopsies) were collected by health professionals in appropriate containers and transported to the BRO Biobank as quickly as possible in a cooled container by the BRO Biobank staff. At the BRO Biobank's facility, the samples were kept in the refrigerator until they were registered in the BRO Biobank. For each donor, a code was given to identify all types of his biological samples and a file was created to keep his signed informed consent and all his medical and personal information.

### 2.5. Processing of Tissue Samples

Tissue samples (surgical specimens or biopsies) were collected by healthcare professionals either in a tube containing the homemade stabilizing reagent “RNAlater” or an empty tube. They were then transported to the BRO Biobank's facility within less than one hour. Upon arrival, tissue samples were recorded in the BRO Biobank and then stored at -80°C.

### 2.6. Preparation of Plasma from Blood Samples

To prepare plasma, the blood samples were collected in EDTA tubes and then transported at 4°C to the BRO Biobank's facility. Within one hour of receiving the blood samples, the plasma was separated from the whole blood by centrifugation at 1800 × g for 15 minutes at +4°C. For each tube, after centrifugation, the upper phase, which corresponds to the plasma, was aspirated and aliquoted into labelled storage tubes (500 *μ*l/tube) and stored at -80°C.

### 2.7. Preparation of Serum from Blood Samples

Blood samples intended for the preparation of serum were drawn in dry tubes. The tubes were inverted 8 times immediately after blood draw to ensure efficient coagulation and were then transported at room temperature to the BRO Biobank's facility. The tubes were centrifuged at 1500 × g for 15 minutes at room temperature, and then, the serum was aspirated and aliquoted into labelled storage tubes (500 *μ*l/tube) and stored at -80°C.

### 2.8. Preparation of White Blood Cells (WBC) from Blood Samples

To prepare WBC, blood samples were collected in EDTA tubes, transported at +4°C to the BRO Biobank's facility, and processed within one hour of blood draw. Thus, after arrival to the BRO Biobank's facility, tubes were centrifuged at 1800 × g for 15 minutes at +4°C. For each tube, the buffy coat layer containing most of the WBC was aspirated and then transferred into 2 ml tubes. To remove excess red blood cells, the WBCs were washed by adding 3 volumes of the TE buffer (20 : 5) and then centrifugation at 2800 × g for 15 minutes at +4°C. Then, 10 volumes of a homemade RNAlater reagent was added to the WBC and the mixture was incubated at +4°C overnight. The following day, 1 volume of PBS was added and then, the tube was centrifuged at maximum speed (5000 g) for 10 min at room temperature. The supernatant was removed, and the WBCs were stored at -80°C.

### 2.9. DNA Extraction from Blood Samples

Blood samples intended for DNA extraction were drawn in EDTA tubes, kept at a temperature between 4°C and 25°C, and processed within 24 hours of blood draw. In the case where the blood samples cannot be processed for DNA extraction within this period, they have been stored at -20°C until use. DNA extraction was carried out using the phenol/chloroform method as described [26]. DNA was aliquoted into labeled storage tubes and stored at -20°C.

### 2.10. RNA Extraction from Blood Samples

To extract RNA, blood samples were collected in EDTA tubes and transported at +4°C to the BRO Biobank's facility. The blood samples must be processed within one hour of blood draw, otherwise RNA will be rapidly degraded because they are very unstable. Thus, upon arrival at the BRO Biobank's facility, the tubes were immediately centrifuged at 1800 × g for 15 minutes at +4°C. For each tube, the buffy coat layer was aspirated and washed by adding 3 volumes of TE buffer (20 : 5) and then centrifugation at 2800 × g for 15 minutes at +4°C. Then, the pellet containing the WBCs was resuspended in 1 ml of “TRizol” reagent and the RNAs were isolated using “TRizol” reagent (Invitrogen). If RNA extraction cannot be performed immediately, the mixture of WBC plus “TRizol” reagent can be stored at -80°C for 15 days until use. After RNA isolation, RNA is aliquoted into labeled storage tubes and stored at -80°C.

### 2.11. Quality Control Analysis of DNA Samples Preserved in the BRO Biobank

BRO Biobank quality control was carried out every 6 months. 5% of tissue samples and 5% of DNA samples of the BRO Biobank were randomly selected and used to assess the DNA quality. Several methods have been used for quality control analysis of DNA samples stored in the BRO Biobank: The yield and purity of DNA samples were assessed by measuring the optical density (OD_260_/OD_280_) ratio using NanoDrop spectrophotometry (Thermo Fisher Scientific). A DNA sample of good quality should have an OD_260_/OD_280_ ratio between 1.8 and 2.1 [27, 28]. The DNA integrity was evaluated by 0.8% agarose gel electrophoresis, and a quality score was assigned for each DNA sample based on the OD_260_/OD_280_ ratio and the DNA electrophoresis profile as described in [28].

In addition, DNA quality was assessed by enzymatic digestion (*Hind*III enzyme, Thermo Fisher Scientific, Lithuania) followed by gel electrophoresis as described in [29, 30]. DNA quality was also evaluated by PCR amplification of four different regions of the *β*-globin gene using the primers shown in Table 1. Obtaining four amplification bands was the indication of a good-quality DNA [27, 29, 31].

### 2.12. Quality Control Analysis of RNA Samples Preserved in the BRO Biobank

10% of tissue samples and 10% of RNA samples of the BRO Biobank were randomly selected and used for RNA quality assessment. As for DNA, the yield and purity of RNA samples were assessed by measuring the optical density (OD_260_/OD_280_) ratio using NanoDrop spectrophotometry (Thermo Fisher Scientific). A RNA sample of good quality should have an OD_260_/OD_280_ratio < 1.8 [28]. The RNA integrity was evaluated by 1.5% nondenaturing agarose gel electrophoresis followed by analysis of the 28S and 18S ribosomal RNAs [27]. RT-PCR was also used to evaluate the RNA integrity as described [32]. In this assay, a region of the GAPDH (glyceraldehyde-3-phosphate dehydrogenase) gene was amplified using the following primers: GAPDH forward: 5′-CCCATGTTCGTCATGGGTGT-3′ and GAPDH reverse: 5′-ATAGCACCTTCCTGAGTACTGGT-3′.

### 2.13. BRO Biobank Data Management

Three different Excel files were created for data management of the BRO Biobank: the first contains all of the donors' medical and personal information. This file is kept in a secure and locked location and only some laboratory members have access to it. The second file is a copy of the first one but does not contain names, medical record number, or any other information that could identify the donors. In this file, information on participants was made anonymous to protect their identity following the guidelines on research ethics. The third file contains information about all biological samples of the BRO Biobank such as the nature of the sample, concentration, volume, quality, and location of the sample in the freezer. As for the second file, code numbers are used instead of names for identification in this file.

### 2.14. Statistical Analysis

Data collection and calculations were performed with Microsoft Excel software version 2013. For qualitative variables, we calculated the percentage. Quantitative variables were expressed as mean and standard deviation (SD).

## 3. Results

A total of 2446 participants were recruited in the BRO Biobank in a period starting from November 2015 to July 2020. Of those participants, 2013 were individuals from the general population of Eastern Morocco “healthy participants” and 433 were patients. Below are the characteristics of these two groups and the corresponding samples stored in the BRO Biobank.

### 3.1. Characteristics of Healthy Participants Enrolled in the BRO Biobank

**Figure 1:**
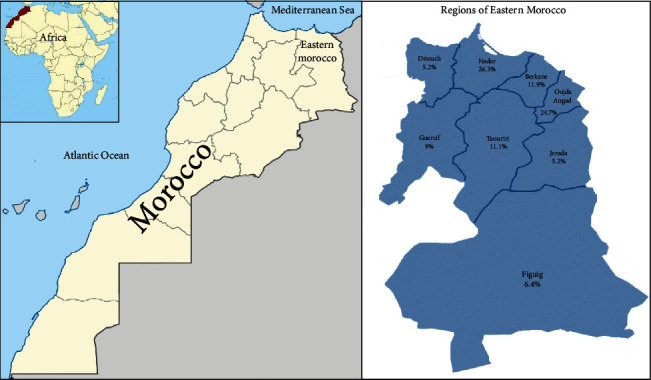
Schematic representation of Eastern Morocco region adapted from “Morocco Map Location” and “Regional Map of Morocco” [33, 34]. The distribution of healthy participants enrolled in the BRO Biobank by region of Eastern Morocco is shown.

**Figure 2:**
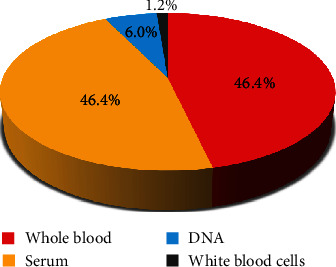
Distribution of biospecimens collected from healthy participants and banked in the BRO Biobank.

Of the healthy participants, 1063 were men and 950 were women, with a male-to-female ratio of 1.12. Participants' median age was 35 years with a range between 18 and 65 years, and the consanguinity rate was 28.96%. Taking into account the percentage of the population living in each region in Eastern Morocco, 530 participants were recruited from Nador, followed, respectively, by Oujda (497), Berkane (241), Taourirt (224), Guercif (182), Figuig (129), Jerada (105), and Driouch (105). The distribution of healthy participants of the BRO Biobank by the region of Eastern Morocco is shown in Figure 1. Biobanking from these participants has resulted in a total of 4341 biospecimens. Of these, 2013 were blood samples, 2013 serums, 261 DNA, and 54 white blood cells (Figure 2).

### 3.2. Characteristics of Patients Enrolled in the BRO Biobank

The personal information of the patients enrolled in the BRO Biobank is described in Table 2. Of the participants, 260 were men and 173 were women, with a male-to-female ratio of 1.50. The median age of participants was 11 years (range 1 days-83 years). In terms of consanguinity, 47% of the patients were from a consanguineous marriage, and of these, 68% were from 1st-degree consanguinity, 20% from 2nd-degree consanguinity, and 12% from 3rd-degree consanguinity. A family history of genetic diseases was found in 36% of the patients. 62% of total cases were recruited in the BRO Biobank in the context of diagnosis and research; in contrast, 38% of cases were enrolled for research only.

**Figure 3:**
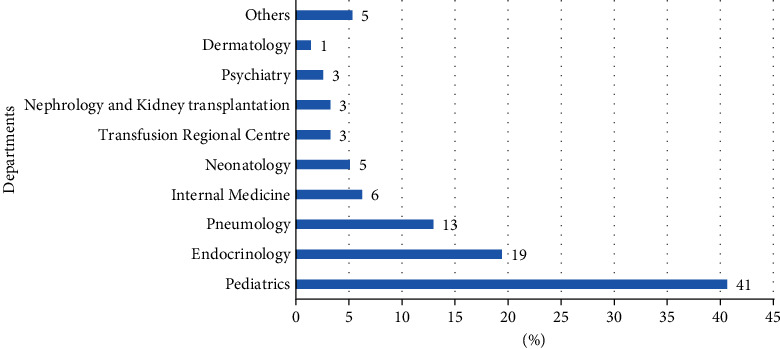
Distribution of biospecimens collected from healthy participants and banked in the BRO Biobank.

The distribution of participants by referral department is illustrated in Figure 3. The majority of patients enrolled in the BRO Biobank were hospitalized in the department of pediatrics (41%), followed by endocrinology (19%), pneumology (13%), internal medicine (6%), neonatology (5%), etc.

Table 3 shows the distribution of patients enrolled in the BRO Biobank by disease type. The most representative diseases were rare diseases (56%), followed by lung cancer (13%), hematological neoplasms (4%), and kidney transplantation (3%). 25% of the patients had unknown diagnoses.

**Figure 4:**
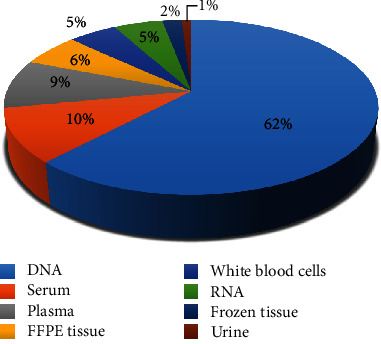
Distribution of collected biospecimens from patients banked by the BRO Biobank.

In July 2020, the BRO Biobank has banked approximately 751 biological samples from patients with various diseases. DNA samples account for 62% of the total number of samples stored in the BRO Biobank, followed by serum (10%), plasma (9%), FFPE tissue (46%), white blood cells (5%), RNA (5%), frozen tissue (2%), and urine (1%) (Figure 4).

### 3.3. Quality Control Assessment of the BRO Biobank

**Figure 5:**
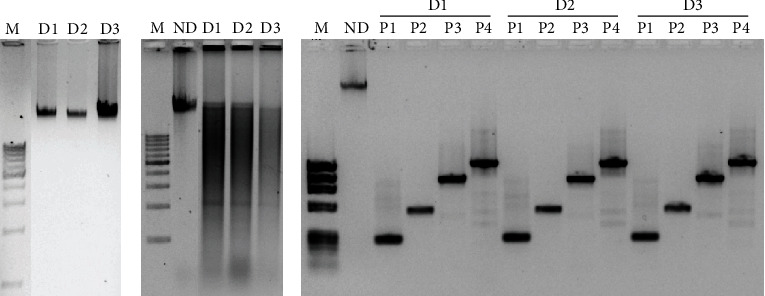
Quality assessment of DNA samples of the BRO Biobank after at least 2 years of storage: (a) 0.8% agarose gel electrophoresis of stored DNA samples; (b) 0.8% agarose gel electrophoresis of DNA samples digested with *Hind*III enzyme; (c) 1.2% agarose gel electrophoresis of PCR amplification of four regions of the *β*-globin gene using DNA samples of the BRO Biobank as templates. M: molecular weight marker (0.1–10 kb); D1, D2, and D3: DNA samples; ND: nondigested DNA; P1, P2, P3, and P4: regions of the *β*-globin gene amplified by PCR.

High-throughput DNA and RNA analysis technologies (exome sequencing, RNA-seq, etc.) require high-quality nucleic acids. Therefore, one of the main challenges of biobanking is to ensure that stored samples are of high quality. Thus, we established several methods for sample quality control of the BRO Biobank and the procedure is described above in Materials and Methods. As an example, Table 4 shows the DNA quality assessment by measuring the ratio OD_260_/OD_280_ of some DNA samples of the BRO Biobank after at least 2 years of storage. The DNA quality assessment of all randomly selected BRO Biobank DNA samples is shown in Supplementary Materials Table S1. All samples showed a ratio between 1.8 and 2.1, confirming the absence of protein contamination and therefore the high purity of these DNA samples [27, 28].  Figure 5 shows the DNA integrity assessment of these samples by gel electrophoresis (Figure 5(a)), enzymatic digestion followed by gel electrophoresis (Figure 5(b)), and PCR (Figure 5(c)). Together, these results suggest that the DNA samples of the BRO Biobank are of good quality even after at least 2 years of storage.

**Figure 6:**
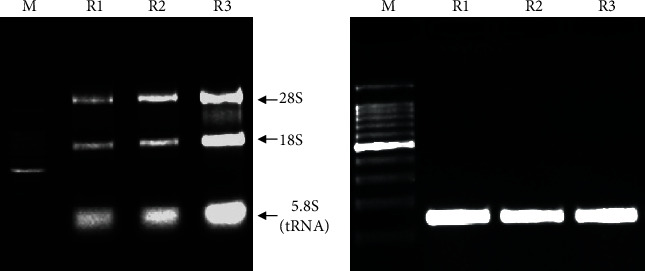
Quality assessment of RNA samples of the BRO Biobank after at least 2 years of storage: (a) 1.5% nondenaturing agarose gel electrophoresis of RNA samples; (b) 2.5% agarose gel electrophoresis of RT-PCR amplification of a region of *GAPDH* gene using RNA samples of the BRO Biobank as templates. M: molecular weight marker (0.1–10 kb); R1, R2, and R3: RNA samples.

As for DNA, RNA quality assessment was undertaken using several methods. Indeed, RNA samples of the BRO Biobank showed a ratio of 1.8, confirming the absence of protein contamination and therefore the high purity of these samples (Table 4). The RNA quality assessment of all randomly selected BRO Biobank RNA samples is shown in Supplementary Materials Table S2. In addition, RNA integrity assessment of these samples by gel electrophoresis (Figure 6(a)) and RT-PCR (Figure 6(b)) showed that they have retained their integrity and therefore are well preserved after at least 2 years of storage.

## 4. Discussion

Biobanks have evolved into complex infrastructures, which significantly contribute to health research and operate at national, regional, and global levels [35, 36]. Indeed, biobanks have accelerated the discovery and development of new treatments by providing sufficient and good-quality samples and data to translational medical research. Therefore, it is extremely important to set up a biobank in every medical research center. For this reason, we launched in November 2015 the BRO Biobank which is, to our knowledge, the first biobank in Morocco.

### 4.1. Impact of the BRO Biobank

There are different types of biobanks. Some biobanks collect samples donated by healthy donors from the general population. This type of biobank is named population-based biobanks and is an essential resource for studies exploring genetic and/or environmental factors implicated in the development of human diseases. These collections require the ability to follow the enrolled individuals over a long time to understand why some individuals develop certain diseases and other do not. China Kadoorie Biobank and UK Biobank are good examples of this type of biobank [37, 38]. Other biobanks focus on a specific disease such as the French glioblastoma biobank, the *β*-thalassemia biobank, chronic kidney disease biobank, and lung cancer biobank, and these types of biobanks are called patient-based biobank or disease-oriented biobank [5, 7, 39–55]. Other biobanks collect samples from patients with different diseases such as the Lausanne institutional biobank and the Japanese biobank [56, 57].

The BRO Biobank has samples from 2013 healthy donors who are representative of Eastern Morocco and can, therefore, be used to further our understanding of the factors that predispose this population to different diseases. Indeed, this population-based collection of the BRO Biobank will allow the long-term study and monitoring of healthy individuals in order to follow the natural development of various diseases in Eastern Morocco population. Furthermore, this cohort is very important in the frequency determination of carriers of autosomal recessive disorders, which are very common in Eastern Morocco due to the high prevalence of consanguineous marriage [58].

In addition to samples from healthy donors, the BRO Biobank harvests and stores samples from patients with various diseases. The majority of these samples are from patients with rare diseases. In fact, during the last five years, our Genetics Unit was the only laboratory in Eastern Morocco offering cytogenetic testing for constitutional chromosomal abnormalities and molecular testing for selected single-gene disorders resulting in availability of many research samples during patient's routine clinical care. Examples of this type of samples that were banked include hemoglobinopathies, syndromic intellectual disabilities, disorders of sex differentiation, and many other genetic conditions. It is worth noting that the rate of consanguineous marriages is still very high in Morocco and therefore, human genetic disorders are so widespread, but they remain uncharacterized [59, 60]. Indeed, research resources in Morocco have been limited and thus, human genetic disorder studies face overwhelming challenges [61, 62]. Consequently, the BRO Biobank is of a great value because it offers unique advantages in studying consanguineous families and in identifying genes that are associated with human genetic disorders in Morocco. Identifying these genes could enable disease recognition and prevention, through genetic screening, which can greatly reduce disease severity as well as the impact and cost to society.

In addition to samples from patients with rare diseases, the BRO Biobank collected samples from patients with various diseases such as kidney diseases, lung cancer, myeloproliferative neoplasms, and other conditions.

Together, the BRO Biobank is both a population-based biobank and a disease-oriented biobank. It is therefore a valuable resource for advancing personalized medicine in many diseases. It also offers the opportunity to study the causes of diseases affecting the population of Eastern Morocco.

### 4.2. Ethical and Legal Issues of the BRO Biobank

In Morocco, there is currently no specific regulation for the creation or activities of biobanks. Therefore, the BRO Biobank was established in accordance with regulations on biobanks already implemented in various countries as well as with the Moroccan law on the protection of individuals with regard to the processing of personal data [20–25]. Therefore, this work will help the creation of other biobanks in Morocco in accordance with ethical rules.

### 4.3. SOPs, Quality Control, and Data Management of the BRO Biobank

Driven by the concern over research reproducibility, biobanks must follow standard operating procedures (SOPs) from sample collection to sample distribution. Thus, our first goal was to adopt good laboratory practices and a rigorous quality control system. Indeed, we have concentrated our effort on developing sample collection, processing, and storage procedures. Therefore, our team composed of highly trained molecular biologists was able to build standards and guidelines in accordance with SOPs that have been established by various biobank organizations, such as the International Society for Biological and Environmental Repositories [9–15]. However, so far, our SOPs have not had any accreditation or certification from any biobanking accreditation system. Therefore, our next step is to achieve accreditation for the BRO Biobank by an official accreditation body. In fact, the granting of accreditation will give confidence to users of biobank samples and facilitate the increasing interinstitutional use of research materials from biobanks [63].

Sample quality control is another very important concern of biobanking. Therefore, we have established several methods for sample quality control of the BRO Biobank in accordance with international guidelines on biobanking [64–68]. DNA integrity following extraction from blood and tissue samples was evaluated, before and after long storage, by gel electrophoresis, enzymatic digestion, and PCR. Similarly, RNA integrity was evaluated by gel electrophoresis and RT-PCR. Furthermore, purity of DNA and RNA isolated from different biospecimens was determined using NanoDrop spectrophotometry. Overall, our results suggested that samples of the BRO Biobank are of high quality and therefore suitable for high-throughput nucleic acid analysis.

In addition to collecting and storing samples, biobanking also involves collecting, managing, and protecting patients' personal and clinical data. Indeed, biobanks require IT and data management systems for tracking and monitoring sample locations as well as for managing patients' personal information [14, 19]. However, due to lack of resources, data management of patients' personal information and samples' location in the BRO Biobank were done simply using Microsoft Excel. Therefore, it is extremely important to acquire IT and data management systems for the BRO Biobank, as the number of samples is increasing and data management is becoming more and more complicated.

### 4.4. Challenges of the BRO Biobank

The major challenge of the BRO Biobank is the involvement of physicians and surgeons in the biobanking process. Indeed, health professionals play a key role in the recruitment of patients into biobanks. We have recently assessed the knowledge and attitude of health professionals in Eastern Morocco towards biobanks and their willingness to recruit patients into biobanks. We found that our health professionals showed a notable lack of knowledge about biobanks. However, the majority were willing to donate their own biospecimens and supported the recruitment of patients into biobanks [69]. We have also evaluated the knowledge of our patients and their attitudes towards biobanks and the reasons that motivate them to participate in biobanks. We found that the majority of our patient expressed their willingness to participate in biobanking through donation of biospecimens associated with personnel and health data [70]. These studies were good opportunities to raise awareness among health professionals and patients about the interest of the BRO Biobank in the development of biomedical research in Eastern Morocco.

Another major challenge we encountered during biobanking was complete data collection of the patient's demographic and clinical information. This task also depends on the involvement of physicians and surgeons in the biobanking process. Therefore, a big effort is needed to raise awareness among health professionals about the importance of complete data from patients to the success of biobanking. In addition, special measures need to be taken for the data collection of the BRO Biobank, which can in fact be integrated into the patient's routine clinical care.

## 5. Conclusion

The BRO Biobank is the largest sample collection in Morocco and is ready to distribute samples to national and international researchers. Therefore, the BRO Biobank is a valuable resource for advancing translational research and personalized precision medicine in North Africa.

## Figures and Tables

**Table 1:** List of primer sequences used for PCR analysis of the *β*-globin gene.

Primer name	Sequence (5′⟶3′)	Size of the PCRproduct in bp
GH20	GAAGAGCCAAGGACAGGTAC	268
PC04	CAACTTCATCCACGTTCACC	
RS42	GCTCACTCAGTGTGGCAAAG	536
KM29	GGTTGGCCAATCTACTCCCAGG	
RS40	ATTTTCCCACCCTTAGGCTG	989
RS80	TGGTAGCTGGATTGTAGCTG	
KM29	GGTTGGCCAATCTACTCCCAGG	1327
RS80	TGGTAGCTGGATTGTAGCTG	

**Table 2:** Characteristics of patients enrolled in the BRO Biobank.

Patients' personal information	*N* (%)
Gender (*N* = 433)	
Female	173 (40%)
Male	260 (60%)

Age	
Median	11 years
Range	1 day–83 years

Consanguinity (*N* = 274)	
Yes	130 (47%)
No	144 (53%)

Degree of consanguinity (*N* = 130)	
1^st^ degree	89 (68%)
2^ed^ degree	26 (20%)
3^rd^ degree	15 (12%)

Family history of genetic diseases (*N* = 273)	
Yes	98 (36%)
No	175 (64%)

Purpose of enrollment (*N* = 433)	
Diagnosis and research	268 (62%)
Research only	165 (38%)

**Table 3:** Distribution of patients enrolled in the BRO Biobank by disease type.

Disease type	*N* (%)
Rare diseases	241 (56%)
Hemoglobinopathy	48 (11%)
Intellectual disability	47 (11%)
Disorders of sex differentiation	32 (7%)
Myopathy	30 (7%)
Multiple endocrine neoplasia	16 (4%)
Genodermatosis	11 (3%)
Metabolic disease	9 (2%)
Autism spectrum disorders	6 (1%)
SMA	5 (1%)
Others	37 (9%)
Lung cancer	56 (13%)
Hematological neoplasms	16 (4%)
Kidney transplantation	13 (3%)
Unknown diagnosis	107 (25%)

**Table 4:** Quality assessment of some DNA and RNA samples of the BRO Biobank after at least 2 years of storage using NanoDrop spectrophotometry.

Samples	D1	D2	D3	R1	R2	R3
Concentration (ng/*μ*l)	145.8	51	318.7	246.9	266	261.4
OD_260_/OD_280_	1.91	1.99	1.96	1.8	1.8	1.8

D1, D2, and D3: DNA samples; R1, R2, and R3: RNA samples.

## Data Availability

Researchers wishing to use the clinical data and/or biological materials of the BRO Biobank have to complete a request form. If their request is accepted by the scientific board, a material transfer agreement is signed and the data and biological materials can be supplied.

## Abbreviations

BRO Biobank: Eastern Morocco biobank
FFPE: Formalin-fixed, paraffin-embedded
PBS: Phosphate-buffered saline
RNAlater: RNA-stabilizing buffer
SOP: Standard operating procedure
TE buffer: Tris-EDTA buffer
WBC: White blood cells

## Supplementary Materials

Supplementary Materials Table S1: quality assessment of DNA samples of the BRO Biobank using NanoDrop spectrophotometry. Table S2: quality assessment of RNA samples of the BRO Biobank using NanoDrop spectrophotometry.
