# Supplementary material for: Morocco's First Biobank: Establishment, Ethical Issues, Biomedical Research Opportunities, and Challenges
Source: Biomed Res Int. 2020 Dec 8;2020:8812609. doi: 10.1155/2020/8812609 (PMC7738781; doi:10.1155/2020/8812609)
Supplement: Supplementary Materials — Table S1: quality assessment of DNA samples of the BRO Biobank using NanoDrop spectrophotometry. Table S2: quality assessment of RNA samples of the BRO Biobank using NanoDrop spectrophotometry. [file 8812609.f1.pdf]

Table S1 : Quality assessment of DNA samples of the BRO Biobank using NanoDrop spectrophotometry

	DNA samples	Concentration (ng/µl)	OD_{260}/OD_{280}
**Quality assessment after 2 years of storage**	**E1**	60.8	1.9
	**E2**	213.1	1.9
	**E3**	290.4	1.9
	**E4**	183.7	1.9
	**E5**	275.2	1.9
	**E6**	243.9	2.1
	**E7**	347.3	2.0
	**E8**	163.1	1.9
	**E9**	247.8	2.0
	**E10**	84.3	1.9
**Quality assessment after 2.5 years of storage**	**E11**	144.5	1.9
	**E12**	125.4	2.0
	**E13**	150.2	2.0
	**E14**	178.2	2.0
	**E15**	225.3	2.0
	**E16**	240.2	2.0
	**E17**	228.5	1.9

	**E18**	155.6	1.9
	**E19**	134.1	1.9
	**E20**	151.3	1.9
	**E21**	187.8	1.9
	**E22**	313.3	1.9
	**E23**	145.8	1.9
	**E24**	51.0	2.0
	**E25**	318.7	2.0

Table S2 : Quality assessment of RNA samples of the BRO Biobank using NanoDrop spectrophotometry

	**RNA samples**	**Concentration (ng/µl)**	**OD_{260}/OD_{280}**
**Quality assessment after 2 years of storage**	**E1**	246.9	1.8
	**E2**	266.0	1.8
	**E3**	261.4	1.8
**Quality assessment after 2.5 years of storage**	**E4**	182.9	1.8
	**E5**	149.9	1.8
	**E6**	186.6	1.7
	**E7**	239.3	1.8
